# Colon Tumor Discrimination Combining Independent Endoscopic Probe-Based Raman Spectroscopy and Optical Coherence Tomography Modalities with Bayes Rule

**DOI:** 10.3390/ijms252413306

**Published:** 2024-12-11

**Authors:** David L. Vasquez, Calvin Kreft, Ines Latka, Jürgen Popp, René Mantke, Iwan W. Schie

**Affiliations:** 1 Leibniz-Institute of Photonic Technology (Leibniz-IPHT), Albert-Einstein-Str. 9, 07745 Jena, Germany; davidl.vasquez-pinzon@leibniz-ipht.de (D.L.V.); ines.latka@leibniz-ipht.de (I.L.); juergen.popp@leibniz-ipht.de (J.P.); 2 Department of Medical Engineering and Biotechnology, University of Applied Sciences—Jena, Carl-Zeiss-Promenade 2, 07745 Jena, Germany; calvin.kreft@eah-jena.de; 3 Institute of Physical Chemistry (IPC), Abbe Center of Photonics (ACP), Friedrich-Schiller-University Jena, Helmholtzweg 4, 07743 Jena, Germany; 4 Clinic for General and Visceral Surgery, University Hospital Brandenburg an der Havel, Brandenburg Medical School, 14770 Brandenburg an der Havel, Germany; mantke.mhb@uk-brandenburg.de; 5 Faculty of Health Sciences Brandenburg, Brandenburg Medical School, 14770 Brandenburg an der Havel, Germany

**Keywords:** colon cancer, Raman spectroscopy, optical coherence tomography, OCT, fiber-optic probes, clinical application, biophotonics, invaScope

## Abstract

Colorectal cancer is one of the most prevalent forms of cancer globally. The most common routine diagnostic methods are the examination of the interior of the colon during colonoscopy or sigmoidoscopy, which frequently includes the removal of a biopsy sample. Optical methods, such as Raman spectroscopy (RS) and optical coherence tomography (OCT), can help to improve diagnostics and reduce the number of unnecessary biopsies. For in vivo use, we have developed fiber-optic probes, one for single-point Raman measurements and one for volumetric OCT. Here, we present the results of a clinical study using these fiber-optic probes in an ex vivo setting. The goal was to evaluate the beneficial effect of combining these two modalities on the AUC ROC score of the machine learning models for the discrimination of cancerous and healthy tissue. In the initial stage of the investigation, both modalities were validated separately using linear discriminant analysis. RS was subjected to spectral preprocessing, while OCT underwent texture feature extraction. Subsequently, both modalities were integrated using the Bayes rule, resulting in an enhanced area under the curve score of 0.93, representing an improvement over the 0.77 score for Raman spectroscopy and 0.86 for OCT.

## 1. Introduction

In 2022, colorectal cancer was the prevalent form of cancer globally, with 1,926,425 new cases reported; 904,019 deaths; and a 5-year prevalence of 5,767,781 cases [1]. The World Health Organization [2] expects that by 2040, the number of cases will increase by 63%, i.e., 3.2 million new cases per year, with a 73% increase in the number of annual deaths, corresponding to 1.6 million deaths per year. The risk factors for this type of cancer are multifold and include modifiable factors, such as alcohol consumption, smoking, sedentary lifestyle, psychological stress, and non-modifiable factors, such as age, gender, genetic predisposition, and intestinal microbiota [3]. Malignant colorectal tumors (carcinomas) arise from pre-existing benign polyps called adenomas, involving the combination of mutational activation mechanisms such as chromosomal instability (CIN), CpG island methylator phenotype (CIMP), and microsatellite instability (MSI) [4,5]. Adenomas develop when the mechanisms that regulate DNA repair and cell proliferation are altered. Polyp adenomas had the potential to increase in size, develop dysplastic features, and acquire invasive potential [6]. The main screening methods for the diagnosis and treatment of adenomas are fecal occult blood tests using the fecal immunochemical test for human hemoglobin and sigmoidoscopy/colonoscopy [3]. Previous studies suggested that flexible sigmoidoscopy reduced the risk of developing distal colorectal cancer by approximately 70% while colonoscopy reduced the overall incidence of colorectal cancer by enabling the removal of precancerous polyps [7]. Adenoma detection rate (ADR) is a widely accepted predictor for endoscopy quality and the endoscopist’s ability to find adenomas and remove pedunculated polyps and large sessile polyps [8]. ADR varies between endoscopists due to the subjectivity involved in the decision process, highlighting the need for image-enhanced modalities to improve the quality of endoscopic procedures and reduce endoscopist-related issues.

Over the last two decades, new optical-based modalities have been developed that go beyond conventional brightfield endoscopy. These methods can provide volumetric, molecular, and metabolic information about a sample, which can significantly improve the diagnostic performance. Modalities such as Raman spectroscopy (RS) provide biochemical information from biological tissues [9]. The inelastic scattering process between a photon and a molecule provides label-free information about the vibrational modes of the molecules under investigation, also known as molecular fingerprints. Numerous studies have demonstrated the ability of RS to improve the detection and diagnosis of cancerous tissue [10,11,12]. However, a common drawback of RS is its low acquisition speed, which makes it less suitable for imaging. We have recently provided a comprehensive review of the multimodal combinations of RS with other modalities [12]. One of the most beneficial combinations is with optical coherence tomography (OCT) [13,14]. OCT is a label-free modality capable of visualizing the volumetric structural information of biological samples at µm spatial resolutions. The contrast mechanism is based on the interference of a reference beam with backscattered light, which occurs at sites with sudden changes in refractive index, such as at boundaries between different tissue types. For example, collagen structures, with step changes within the tissue, provide optimal contrast. This modality has been used to detect tumor borders and to improve the extraction of tumor tissue in different types of cancer, including skin cancer, head and neck carcinomas, lung cancer, breast cancer, colorectal cancer, prostate, renal, ovarian, and bladder cancer [15].

There are significant advantages to combining multiple optical modalities, as they provide complementary information that can significantly advance the understanding of a disease and improve the diagnostic accuracy. Therefore, there has been considerable interest in combining multiple modalities in recent years [12,16,17,18]. The characterization of colorectal tumor tissue using OCT and RS has readily been reported by Ashok et al. [19] using a microscopy-based system. The results indicate that normal and tumor tissues can be differentiated with a sensitivity and specificity of 94%. While the results are very encouraging, the clinical application of a microscopy-based system has limited suitability for in vivo clinical applications due to the sheer size limitation of the system. To enable a clinical translation, the implementation of both modalities into fiber-optical probes is required. Nevertheless, fiber-optical probes commonly lack the performance of microscopy systems due to the miniaturized implementation of the optical and mechanical systems in these probes. We have previously reported on the development of fiber-optic probes for Raman spectroscopy and OCT and the application of the fiber probe-based characterization of bladder cancer in ex vivo and in vivo studies [20].

In this work, we evaluate the suitability of using fiber-optic probes for Raman spectroscopy and OCT for the diagnostic characterization of ex vivo colorectal cancer biopsies. This is a necessary step for the in vivo translation of endoscopy-based systems. We have developed a data processing pipeline for each individual modality and for the combination of both modalities using the Bayes rule. For the OCT data analysis, we use features extracted from texture analysis, while for the RS, we work with preprocessed spectra. We have built machine learning models to evaluate and validate the performance of classification algorithms using linear discriminant analysis (LDA). Our results indicate that by combining the multimodal information in our framework, we can achieve an increase in the receiver operating characteristic (ROC) area under the curve (AUC) score from 0.77 for Raman spectroscopy and 0.86 for the OCT to a combined score of 0.93. This demonstrates that there is an increase in the accuracy of the classifier when multimodal information is used.

## 2. Results and Discussion

In this study, 90 colon polyp samples from 30 subjects were collected at the University Hospital of Brandenburg an der Havel. The study was approved by the Ethics Committee Brandenburg Medical School (E-02-20210414). After the removal of the tumor specimen of colorectal carcinoma of all stages, they were cryopreserved at −80 °C and later characterized using OCT imaging and RS. For correlation, healthy tissue from colon mucosa was also extracted and characterized. Because the quality of Raman spectra was poor in some of the measurements, for further analysis, a total of 61 samples (21 healthy/40 tumor), and 303 Raman (132 healthy/171 tumor) and 346 OCT (144 healthy/202 tumor) measurements were used (see Table 1).

**Figure 1:**
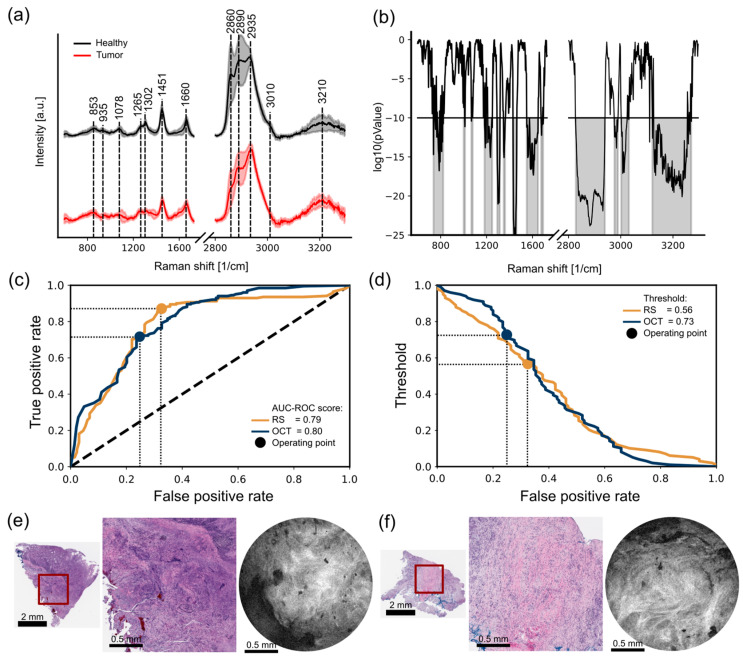
(**a**) Mean healthy spectra and mean tumor spectra with ±1 standard deviation. Representative biomolecule bands are highlighted. (**b**) *p*-value result from Welch’s test; bands with a statistical relevance of values < 10^−10^ are highlighted and used as the input dataset for the RS LDA classifier. (**c**) ROC curves for the RS and OCT LDA classifiers showing the optimal operating point adopting the Youden index. (**d**) FPR vs. threshold plot showing the corresponding threshold related to the optimal operating point; (**e**,**f**) are two tumor samples with dense collagen microstructures characteristic of desmoplastic tumor patterns and the corresponding OCT enface image. The red box highlights the zoomed-in area of the histological slide, showing an equivalent field of view.

To achieve molecular characterization using the endoscopic Raman probe, multiple Raman spectra were acquired in different positions pressing the endoscopic probe against the samples and emulating a clinical procedure. After recording the raw Raman spectra, the entire dataset was preprocessed as a single batch (see Section 3.4). Once the background contributions were removed, the mean spectra for the healthy and tumor samples were plotted together with a ±1 standard deviation (see Figure 1a). In the mean spectra, distinct vibrational bands associated with common biomolecular bonds, e.g., proteins and lipids, were identified. The bands at various wavenumbers are explained in the following: 853 cm^−1^ is related to proline, hydroxyproline, and tyrosine; 935 cm^−1^ for the C-C stretching of proline; 1078 cm^−1^ is related to the Alkyl C-C gauche stretches, which is common in lipids; and 1265 cm^−1^ and 1661 cm^−1^ are related to secondary structural information, specifically Amide III and Amide I, but also to common bands present in lipids, i.e., Alkyl C-H cis stretch and Alkyl C=C stretch. Additionally, peaks at 1302 cm^−1^ for CH_2_ twisting and 1451 cm^−1^ for CH_2_ and CH_3_ deformation were identified and related to protein and lipid alike. In the high-wavenumber region, peaks were detected at 2860 cm^−1^ for the CH_2_ symmetric stretch of lipids, 2890 cm^−1^ for the CH_2_ asymmetric stretch, 2935 cm^−1^ for the CH_3_ symmetric band, 3010 cm^−1^ for the unsaturated =CH stretch, and 3210 cm^−1^ for water OH-stretching and NH-stretching. All the discussed Raman peaks are listed in Table 2.

To calculate the statistical significance of each wavenumber band, the Welch’s test or unequal variance *t*-test was performed (see Figure 1b). *p*-values below 10^−10^ were selected and used for further analysis in the classification phase. In total, 13 regions were selected: 737–822 cm^−1^, 1000–1012 cm^−1^, 1067–1079 cm^−1^, 1182–1249 cm^−1^, 1293–1315 cm^−1^, 1349–1361 cm^−1^, 1431–1461 cm^−1^, 1550–1648 cm^−1^, 1676–1693 cm^−1^, 2828–2939 cm^−1^, 2976–2989 cm^−1^, 3002–3031 cm^−1^, and 3121–3270 cm^−1^.

An LDA classifier for the diagnosis of healthy and tumor samples was validated based on the relevant spectral regions. A nested leave-one-subject-out cross-validation (CV) was implemented to estimate the classifier’s performance while maximizing the amount of training data and reducing the overfitting. For the details of the implementation of the RS model validation, see Section 3.5.

The selected merit function to compare the performance of the LDA classifiers is the AUC-ROC, where the false positive rate (FPR or 1-specificity) versus the true positive rate (TPR or sensitivity) is plotted for a given binary classification task. In this curve, all the possible combinations of FPR and TPR that the classifier is capable of achieving are integrated. The resultant ROC curve for the RS LDA classifier along with the AUC-ROC score of 0.79 are shown in Figure 1c. The thresholds associated with the corresponding ROC curves are shown in Figure 1d.

The Youden index, a metric designed to maximize the sum of sensitivity and specificity, was selected to determine the optimal operating point. For the RS LDA classifier, the optimal operating point is FPR = 0.33, TPR = 0.87, and a decision threshold of 0.56, as highlighted in Figure 1c,d. The confusion matrix for the optimal operating point is summarized in Table 3.

Classification between colorectal tumors and healthy samples using OCT images is based on the characteristic changes in features linked with the presence of tumor, such as desmoplastic patterns associated with the development of fibrosis tissue around tumor cells [23]. In Figure 1e,f, two tumor samples, displaying dense collagen fibers, are presented. These features are well suited for pattern recognition. Four feature extraction methods were selected for this study, which are based on texture descriptors for texture analysis, i.e., Gabor filters, local binary patterns, local phase quantization, and gray-level co-occurrence matrix. These methods ensure invariance to size, shape, and orientation (see Section 3.9). The texture features are extracted from gray-scale OCT images and are used to train and test an independent LDA classifier. The OCT image dataset consists of enface images obtained from OCT endoscopic probe measurements within the tissue. These images have a 2 mm field of view with a pixel size of 4 μm (see Section 3.7).

The structure of the OCT model validation differs from the RS algorithm in how PCA coefficients are concatenated before the validation phase during the training and model implementation during testing. Since four distinct texture features are used, normalization and standardization are performed separately for each feature, and the results are concatenated after calculating the PCA coefficients (see Section 3.9). The leave-one-subject-out CV, hyperparameter tuning phase, AUC-ROC merit function, and Youden index stay the same for both RS and OCT model validations. The results from the OCT LDA classifier are presented in Figure 1c,d. An AUC-ROC score of 0.80, with an optimal operating point of point FPR = 0.25, TPR = 0.72, and decision threshold = 0.73, is achieved. The performance of both classifiers is similar in terms of the AUC-ROC scores, with slightly better performance for the OCT classifier. A difference in the decision threshold is observed for the optimal operating point between 0.56 for RS and 0.73 for the OCT classifier.

**Figure 2:**
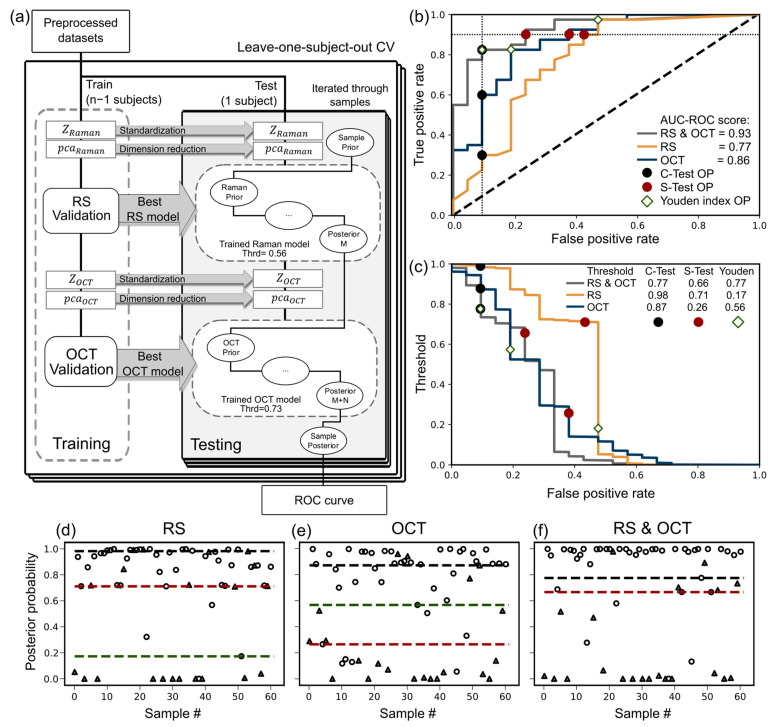
(**a**) Model validation algorithm to assess the performance of the Bayes classification method combining the RS and OCT endoscopic probe measurement. (**b**) ROC curves of model validation for RS (yellow), OCT (blue), and the combined RS and OCT (gray) modalities. The operating points were chosen based on the Youden index (diamonds), the confirmatory test (black circles) and the screening test (red circles). (**c**) corresponds to the optimal decision threshold for the posterior tumor probabilities, (**d**) the posterior probabilities of the Bayes classification method using only RS, (**e**) using only OCT, and (**f**) using the combination RS and OCT. The dashed lines represent decision thresholds, and the triangles and circles represent the healthy and tumor samples, respectively.

To integrate the measurements recorded by the RS and OCT endoscopic probes and develop a tumor tissue diagnosis algorithm, a combined classification method based on Bayes’ rule was implemented. The algorithm used for this purpose and the corresponding model validation are shown in Figure 2a. The preprocessed dataset was split following the leave-one-subject-out CV algorithm. During the training phase, standardization, dimension reduction, and the best-performing model were calculated for both modalities according to the steps outlined previously in Figure 1. In the testing phase, the posterior tumor probability is calculated over each sample in the test subject. The starting prior probability for each sample was set to 0.5. The RS test set was standardized and reduced in dimension. Using the best RS model from the training phase, the prior sample probability was updated M times, where M is the number of RS records in each sample applying the Bayes rule. The decision threshold for the best RS model was determined in the first part of the study, threshold of 0.56, identified as the optimal value for the RS model. It is important to note that the RS and OCT models may differ in their ROC curve shapes across iterations. However, to ensure consistency, the same decision threshold is used throughout all the iterations. Next, the OCT test set records were standardized and reduced in dimension. The best OCT model from the training phase is used to update the prior probability N times, where N is the number of OCT records in the corresponding sample. The tumor decision threshold is set to 0.73 following Figure 1d. The posterior disease probability of the RS modality was used as the prior probability for the OCT modality. At the end of the algorithm, the tumor probability for each sample is calculated exactly once. This enables us to evaluate the performance of the combined Bayes classification model using a single ROC curve and the corresponding AUC-ROC score. Bayes rule is invariant to the order of application, making the sequence of modalities irrelevant. For comparative analysis, the Bayes classification model was also applied to individual modalities, where the algorithm is run exclusively on either the RS or OCT dataset.

The Bayes classification results for RS, OCT, and the combination of both modalities (RS and OCT) are presented in Figure 2b. The results indicated an AUC-ROC score of 0.77 for RS, 0.86 for the OCT, and an improved AUC-ROC score of 0.93, when both modalities were combined.

To evaluate the performance in a specific application of the Bayes classification method, it is necessary to select appropriate operating points. Since the type of application determines the relevant region of the ROC curve, two distinct areas of the curve are analyzed. The first application is the confirmatory test (C-test), which is designed to confirm diseases and is characterized by high specificity (or a low FPR). For this case, operating points at FPR values equal to 0.1 are marked with black dots on the ROC curve (see Figure 2b). Similarly, the screening test (S-test), which aims to detect as many positive cases as possible, is characterized by high sensitivity. A TPR value equal to 0.9 is selected with the corresponding operating points indicated by red dots on the ROC curve. In Figure 2c, the decision thresholds for each operating point are displayed, and the confusion matrices for the individual cases are presented in Table 4. The thresholds are additionally shown in Figure 2d–f, where the posterior probabilities for each sample in the study are plotted for the results of the Bayes method for RS, OCT, and the combined RS and OCT, respectively. The interrupted lines represent decision thresholds, triangles represent healthy samples, and circles represent tumor samples.

It is important to note that, up to this point, the AUC-ROC metric has been used to evaluate the performance of the trained classifiers and the Bayes method. However, for comparing operating points, the accuracy score is used. In Table 4, a comparison of results for the single RS and OCT modalities shows that according to the presented algorithm, both modalities perform better as a screening test than as a confirmatory test. Specifically, the accuracy for the S-test is 0.79 and 0.80, whereas for the C-test, it is only 0.51 and 0.70. The combined RS and OCT results demonstrate a significant improvement in the performance as a C-test, with scores increasing from 0.51 and 0.70 to 0.85. Similarly, as an S-test, there is a moderate improvement in accuracy, with the score rising from 0.79 and 0.80 to 0.85.

A third case is analyzed, where the operating point is selected based on the Youden index. In Figure 2b, these points are represented by a diamond shape. The Youden index operating point aligns with the RS and OCT results for the C-test, suggesting that although the T-test and C-test regions exhibit the same accuracy, the combined RS and OCT is slightly better suited for C-test.

When comparing the Youden operation points, the combined RS and OCT method shows an improvement in accuracy, increasing from 0.82 to 0.85.

## 3. Methods

### 3.1. Tissue Samples

**Figure 3:**
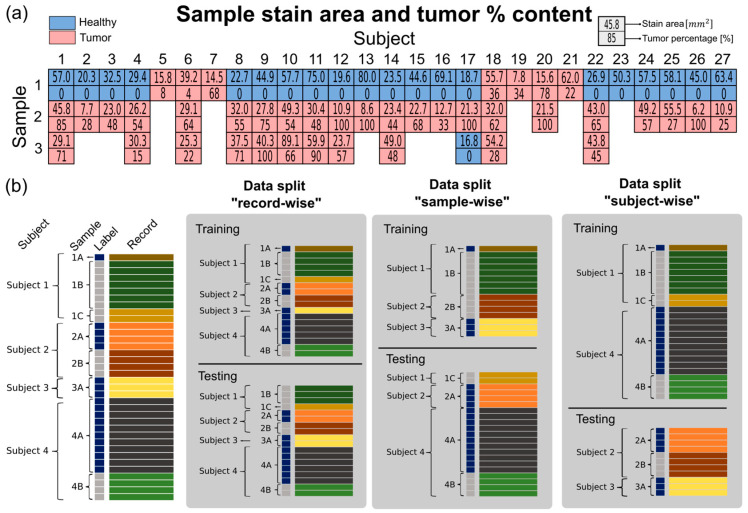
Sample dataset summary. (**a**) A total of 61 tissue samples from 27 patients were used in the analysis. The area of each sample is determined from the H&E-stained and annotated images. The blue color labels the healthy samples and the red color labels tumors. Since the tumor samples could contain mixed regions, i.e., tumor and healthy regions, the area ratio of tumor and healthy tissue was calculated for each individual biopsy. (**b**) Example dataset with 4 subjects, 8 samples, and 36 records showing 50–50% record-wise, sample-wise, and subject-wise data split. For the record-wise data split, it is possible to balance the partitions, while in the other two cases, the balance may vary, potentially causing a data distribution mismatch. In the subject-wise data, split eliminates the issue of identity confounding. In the graph, the label color means blue: healthy and gray: tumor; the record color belongs to a single sample.

Tissue samples were obtained during normal endoscopic procedures targeting healthy, tumor, and tumor border tissue. The study included all the male and female patients diagnosed with colorectal carcinoma who underwent surgery and were willing to provide tissue from the removed tumor for scientific analyses. Participants had to be over 18 years old and able to comprehend the patient information provided. Exclusion criteria included patients who did not meet these criteria or who withdrew their consent to participate. The study did not influence the patients’ therapy, ensuring that the priority remains on obtaining pathological tissue for disease diagnosis. Ex vivo RS and OCT measurements were conducted with endoscopic probes successively on all the samples and on the same day. This ensured that potential system variations could be reduced or avoided. Following the measurements, the samples underwent histopathological processing and a representative slide with the pathologist’s corresponding annotation served as the ground truth for the evaluation. The biopsy samples were not homogeneous, meaning they usually contained both tumor and healthy regions, which is summarized in Figure 3a. The table shows the size of the histologically stained and sliced samples and the corresponding tumor area in percentage. Samples containing any tumor area were labeled as tumor, i.e., areas with a tumor content of more than 0%. The area of the stained images ranged from 7.7 to 89.1 mm^2^ and could be quite heterogeneous, as can be seen from the values. The heterogeneity caused by the presence of both healthy and tumor tissue in tumor samples is an important consideration during data processing, because it increases the variability within the tumor category and reduces the performance of classifiers as we have previously shown [20].

### 3.2. Datasets Split

The highly complex dataset consists of multiple measurements for each sample and each modality. There are several ways to properly handle and analyze the data. Different split methods offer certain advantages and disadvantages, which have been discussed in Tougui et al. [24]. In our setting, three different approaches for train-test splitting the data are possible, i.e., record-wise, biopsy-wise, and subject-wise. Given the limited number of samples available, the advantage of the record-wise approach was the possibility of having balanced training and test sets. In Figure 3b, an example of the dataset, consisting of a total of four subjects, eight samples, and 36 records (or measurements), is shown. In the record-wise case, the number of healthy and tumor samples is balanced between the test and train sets, but records from the same sample could be mixed in both sets. This could lead to “identity confounding” [25] and an overestimation of the model’s performance. The sample-wise split reduces this problem by assigning records from the same sample either to the training or test set. However, the identity confounding is not entirely removed, since there is the possibility to find subjects with only healthy samples. To mitigate this problem, a good practice is to use the highest hierarchical level to split the data [26] which in this case would be the subject-wise split.

### 3.3. Raman Instrumentation

The Raman measurements were performed using the invaScope system reported by Latka et al. [27]. The system is based on a narrow-band continuous wave 785 nm diode laser (Fergy, Princeton Instruments, Trenton, NJ, USA) and an adjustable laser power of up to 500 mW. For the experiments, the nominal excitation power was around 60 mW. The excitation light was coupled in a 3 m long fiber endoscopic probe designed for in vivo applications, featuring one central excitation fiber and ten outer collection fibers with a core diameter of 105 µm and a numerical aperture of 0.22. To remove the Raman background generated in the excitation fiber, a window was placed at the distal tip with two concentric optical filters (Optics Balzers, Jena, Thuringia, Germany), a cleanup filter (785 nm ± 1.5 nm) in the center, and a high pass filter (800 nm) at the border. The outer high pass filter blocked the excitation light and allowed the generated Raman signal from the sample to pass. The Raman scattering occurred isotropically within the sample and was typically collected from depths of down to 500 µm by the collection fibers. Next, an additional notch filter in the invaScope unit (785 ± 10 nm, Semrock, Rochester, NY, USA) suppressed any residual excitation light. The signal was guided to a spectrometer containing a 1200 groves/mm grating, blazed at 870 nm, allowing a spectral resolution of 5 cm^−1^. A charge-coupled device (CCD) (PIXIS100BR eXcelon, Princeton Instruments) recorded the spectra with an active area of 26.9 × 2 mm and of >90% nominal quantum efficiency for the low-wavenumber region between 800 and 910 nm. A calibration process was performed and saved before each sample was measured.

### 3.4. RS Preprocessing

**Figure 4:**
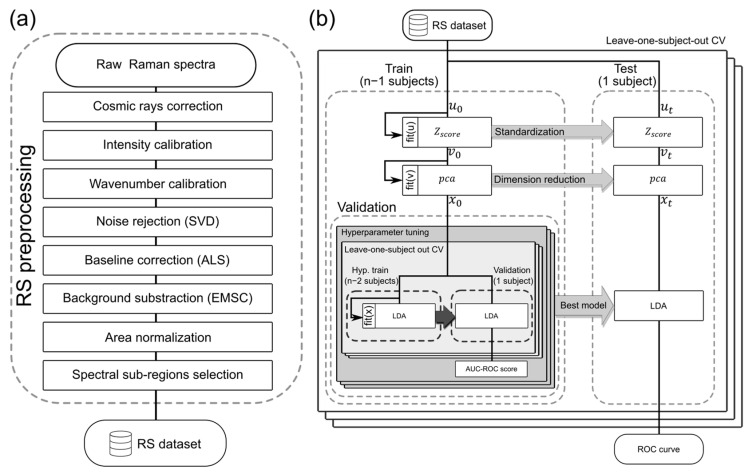
(**a**) step-by-step operations used for the RS preprocessing of the raw Raman data, (**b**) RS model validation algorithm of an LDA classifier for tumor tissue diagnosis. Leave-one-subject-out CV was implemented to assess the classifier's performance. In the training phase, the data were standardized using a Z-score function, and the dimension was reduced using PCA. The validation was built with a hyperparameter tuning loop, a nested leave-one-subject-out CV, and the AUC ROC score as a merit function. In the test phase, the data were standardized and reduced in dimension using the same functions from the training phase. The best score-wise performing LDA model from the validation was used to infer the test set and to report the tumor probability of each record. The ROC curve of the model validation was reported.

Raw Raman spectra typically contain different background contributions and signal modulation, including autofluorescence background, convolution with system response function, and cosmic spikes. To correct for these factors, raw Raman spectra were preprocessed before the implementation of an LDA classifier (see Figure 4a). The cosmic spikes were corrected by using a derivative and interpolation-based algorithm [28]. The spectra were calibrated in the wavenumber axis using a reference spectrum of polystyrene and a fifth-order polynomial function. Furthermore, the system intensity response function was calibrated using the NIST-certified SRM-2241 reference target following established procedures. In the next step, the spectra were corrected for noise contributions by singular value decomposition (SVD), where the first 30 components were retained. The polynomial baseline was removed using asymmetric least squares (ALS) with the parameters lambda = 10^3^, *p* = 10^−5^, within 10 iterations [29]. Additionally, background contributions from known constituents, such as holder material and room light, were removed using extended multiplicative signal correction (EMSC). Consecutively, the Raman spectrum was area normalized, and the relevance of the wavenumber bands was calculated using the Welch *t*-test (or unequal variance *t*-test), where the bands with *p*-values under 10^−10^ were considered statistically significant.

### 3.5. RS Model Validation

The preprocessed RS dataset served as the starting point for the training, validation, and testing of an LDA classifier. The entire process is visualized in Figure 4b. To evaluate the method’s performance, leave-one-subject-out cross-validation was implemented using the ROC curve as the scoring metric. The training set was then normalized using Z-score standardization, where the individual spectra were subtracted by the training set mean value and divided by the training set standard deviation. Subsequently, the normalized set was used for principal components analysis (PCA) and the first ten components of each spectrum were used as an input in the validation loop.

The validation process consisted of a hyperparameter tuning loop, where all combinations of the model’s hyperparameters were evaluated using a grid search, returning the optimal model configuration. The classification task is performed by an LDA model, with the type of solver (singular value decomposition, least square solution, and eigenvalue decomposition) and tolerance (10^−2^, 10^−4^, 10^−6^, 10^−8^, 10^−10^, 10^−12^, and 10^−14^) as the hyperparameters to tune. A nested leave-one-subject-out CV and the AUC-ROC score are used to evaluate the models. The model achieving the best score is stored for later testing.

To maintain consistency in the testing, the test set was Z-score normalized using the parameters calculated from the training set. Similarly, the PCA coefficients were calculated using the principal components derived from the training set. Finally, the best score model was used to evaluate the test data, providing the predicted tumor probability of each test record. As a result, an ROC curve is reported representing the performance of the LDA classifier trained with the RS dataset.

### 3.6. OCT Instrumentation

The OCT system is a spectral domain-based implementation in combination with an endoscopic fiber-based probe. The excitation light consists of three super luminescent diodes, resulting in a center wavelength at 850 nm, a spectral bandwidth of 125 nm, and 10 mW power (cBLMD-T-850-HP-I, Superlum, Carrigtohill, Co. Cork, Ireland). The excitation light was split 50:50 in the reference and sample arm using a standard fiber-coupled splitter. The illumination light in the sample arm was guided to a 3 m long endoscopic fiber probe with a solid distal tip length of 40 mm and 4.5 mm in diameter. The endoscopic probe is based on the scanning fiber principle, where a single cantilevered fiber is excited at the mechanical resonance frequency, producing a growing scanning spiral pattern [30]. In the sample, the illumination light was scattered ballistically due to changes in the microstructure refractive index and was collected by the same scanning fiber. The signal was interfered with the light of the reference’s arm and the resulting interference fringes were recorded by a high-speed spectrometer (Octopus Cobra-S800, CS800-850/140-250-OC2K-CL, Wasatch Photonics, Morrisville, NC, USA). The spectrometer provided a 2.6 mm image depth, a wavelength range from 780 to 920 nm, and a 250 kHz line scan rate. The endoscopic probe provided a field of view of 2 mm, a working distance of 160 µm in air, a spot size with a full width at half maximum (FWHM) of 6.8 µm, and an axial depth FWHM of 3.7 µm in air.

### 3.7. OCT Preprocessing

The raw OCT data were directly obtained from the interferogram in the spectrometer. A single point measurement, referred to as an A-scan, encoded morphological information along the depth axis. These A-scans were captured sequentially, with the measurement point moving in a predetermined spiral pattern controlled by the scanning fiber within the endoscopic OCT probe. The scanning pattern was recorded using a position-sensing detector, and the spatial coordinates were utilized as a lookup table to map the A-scans to their corresponding XY positions within the scanning pattern. After recording an OCT measurement, standard data processing techniques were applied to extract information from the interferogram and convert it into morphological information. For spectral domain OCT, the preprocessing included wavenumber calibration, equidistant data resampling, dispersion compensation [31], background subtraction, high-pass filtering, fast Fourier transform, and logarithmic scaling. At last, the lookup table and A-scans were interpolated onto an evenly spaced rectangular grid, generating a 3D volume that represents the sample morphological structure.

**Figure 5:**
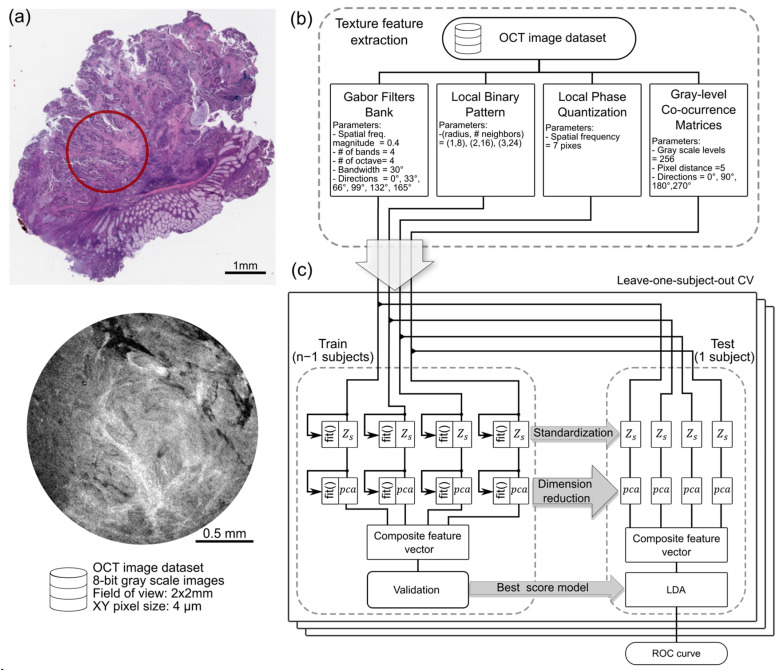
(**a**) H&E-stained tumor sample showing the endoscopic probe OCT measurement field of view (red circle) in relation to the sample and the properties of the OCT image dataset. (**b**) Selected methods for texture feature extraction with the implemented parameters. (**c**) OCT model validation algorithm to assess the performance of an LDA model trained with the OCT image dataset. The algorithm standardizes and reduces the dimension of each texture feature individually to later concatenate the PCA coefficients in a composite feature vector. From this point, the algorithm is the same as the one used for RS model validation.

From the obtained 3D volume, two primary types of 2D images could be extracted: enface images, which display information parallel to the sample surface, and B-scans, which show cross-sectional information perpendicular to the sample surface. In this study, the enface images were used for the analysis due to their larger area for the feature extraction algorithms, as well as isotropic lateral resolution in the image. For further validation, only one representative enface image per OCT measurement was selected. This avoids interrelated or potentially redundant information in the analysis. Depending on the size, multiple measurements were made on each biopsy. In total, this resulted in a set of 346 independent enface images for the evaluation of the LDA classifier (see Figure 5a).

### 3.8. Texture Feature Extraction

Four texture feature extraction methods were selected based on their invariance to size, shape, and orientation. These methods extracted texture descriptors from the images, which were then used as input features for the OCT LDA classifier (see Figure 5b).

The Gabor filter bank extracts information about the spatial frequency and texture orientation from the input image based on the set of implemented filters. The selected filters were generated with a spatial frequency magnitude of 0.4, four bands, one octave, and a 30-degree bandwidth, in six different directions (0°, 33°, 66°, 99°, 132°, and 165°) [32,33]. This configuration allows the filters to capture a wide range of texture orientations and spatial frequencies, enhancing the ability to analyze and distinguish different patterns within the image. Local binary patterns provide information on neighborhood structures, utilizing a sampling scheme for 1-, 2-, and 3-pixel radii with the P numbers of neighbors set to 8, 16, and 24, respectively. The local binary patterns algorithm returns histograms and variances that can be used as sample descriptors [34]. Local phase quantization, which is based on a discrete Fourier transform, preserves invariance to blur by computing phase information in a local neighborhood region of 7 pixels, returning a histogram as a descriptor [35]. The gray level co-occurrence matrix describes the pixel distribution on the texture [36], with matrices computed at a distance of 5 pixels and angles of 0°, 90°, 180°, and 270°. The gray levels were set to 256, and the returned targeted properties included contrast, dissimilarity, homogeneity, energy, correlation, and angular second moment. These calculated features were presented in the form of histograms and vectors and used for further processing steps.

### 3.9. OCT Model Validation

The OCT model validation started with a leave-one-subject-out CV. During the training phase, each feature was normalized based on Z-scores standardization, followed by a dimensionality reduction for each of the four methods (see Figure 5c). Thereafter, the PCA scores of each method were concatenated into a resulting composite feature vector. The set of composite vectors was used in the validation loop, similar to the RS approach, where the LDA model with the best score from the validation loop is selected and stored. During the testing phase, the standardization functions and PCA components generated in the training phase were applied to the test set. The resulting PCA scores were later concatenated into a composite feature vector and used to test the best score LDA model. The algorithm outcome was the ROC curve, representing the performance of the LDA model for the OCT image dataset.

### 3.10. Combination of Modalities Using Bayes Rule

Bayes’ rule is a functional statistical tool used to update probabilities based on new evidence. It is applied across different fields, including medical diagnostics. Given the result of a diagnostic test, it allows us to reassess the probability or uncertainty of a disease, called the posterior probability, starting from an initial probability of a disease, called the prior probability. In our proposed framework described in this manuscript, each RS and OCT measurement is handled as an independent diagnostic test M, where each subsequent result updates the positive probability of the sample being a tumor. One must keep in mind that there can be multiple RS and OCT measurements for an individual biopsy. Here, the posterior probability of disease was calculated using the following two equations: positive predicted value when the diagnostic test is positive (tumor) [37]

(1) $$posterior=\frac{TPR\times prior}{\left(TPR\times prior\right)+\left(FPR\times \left(1-prior\right)\right)}$$

positive predicted value when the diagnostic test is negative (healthy)

(2) $$posterior=1-\frac{\left(1-FPR\right)\times \left(1-prior\right)}{\left(\left(1-FPR\right)\times \left(1-prior\right)\right)+\left(\left(1-TPR\right)\times prior\right)}$$

**Figure 6:**
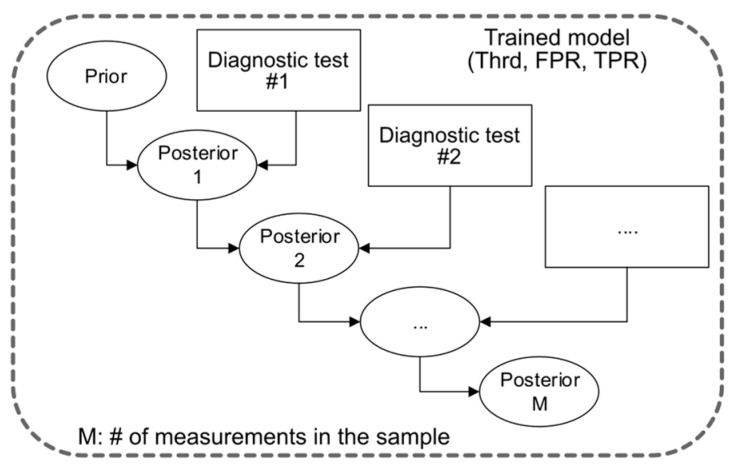
Bayes rule for M diagnostic tests applied to calculate the final posterior probability. The decision threshold (Thrd) defines the operating point on the ROC curve, determining the FPR and TPR. Using this information, along with the diagnostic test result, the posterior probability can be calculated after each test. Here, the diagnostic test M is an individual measurement with either RS or OCT of a sample where multiple measurements could take place per sample.

The application of Bayes’ rule requires the prior probability and the diagnostic test information, including the diagnostic test result (positive/negative), the true positive rate (TPR), and the false positive rate (FPR). If the diagnostic test result was positive, Equation (1) was applied; if the result of the diagnostic test was negative, Equation (2) was applied. Given a trained LDA classifier, the RS and OCT measurements were converted into a diagnostic result. The ROC curve of the trained model provides all the possible combinations of threshold, FPR, and TPR. The optimal operation point was set by the Youden index. Figure 6 shows the algorithm for the calculation of the posterior disease probability of M diagnostic tests with a given trained model. The posterior disease probability was sequentially updated M times. Bayes’ rule is ordering invariant, meaning the order sequence is not relevant.

## 4. Conclusions

The results demonstrate that the individual modalities applied, i.e., RS and OCT, perform reasonably well on their own in an endoscopic fiber probe implementation and achieve moderate accuracy in the diagnostic characterization of ex vivo colorectal cancer biopsies. The mean Raman spectra showed good agreement with the existing studies. The Bayesian classification method successfully combined both modalities, improving diagnostic accuracy and compensating for the limitations observed in the performance of each modality. Specifically, the integration of RS and OCT through Bayes’ rule allowed us to increase the AUC-ROC score from 0.77 (Raman) and 0.86 (OCT) to 0.93 for the combined model. This multimodal approach proved effective despite the individual variability and challenges associated with the separate modalities. In our work, we have set a prior probability of 0.5 for each sample, reflecting an equal uncertainty of being classified as healthy or tumor, with no additional risk factors considered. Consequently, the results are based entirely on the optical modalities information. However, this prior probability could be adjusted in future studies to incorporate additional relevant information, potentially improving the diagnosis accuracy. In the ROC curve of non-ideal diagnostic tests, the operating point must be balanced between high sensitivity (low FPR) and high specificity (high TPR), depending on the application’s requirements. Furthermore, the ROC curves change with each iteration, adding an additional variable to the Bayesian model. To address this, the optimal operating point was determined using the Youden index, providing an optimal decision threshold of 0.57 for RS and 0.73 for OCT. The fixed threshold provided a consistent framework for evaluation across models. This study provides a significant step toward the in vivo translation of endoscopy-based systems by demonstrating the applicability of endoscopic fiber probes for RS and OCT. The data processing pipeline developed for individual and combined modalities with the Bayes rule supports the future implementation of the label-free characterization of colon cancer using these techniques. The results suggest an increase in classifier accuracy when combining multimodal information, paving the way for the future clinical applications of endoscopic fiber systems for the reliable, non-invasive characterization of colorectal cancer. This work sets the stage for future studies where the external validation of the methods and the model approach can be conducted.

## Figures and Tables

**Table 1:** Summary of the subjects, samples, and records used in the study.

**# of subjects**	**# of healthy samples**	**# of tumor samples**	**Total**
27	21	40	61
	**# of healthy records**	**# of tumor records**	**Total**
RS	132	171	303
OCT	144	202	346

**Table 2:** Raman band assignments for vibrational bands which are indicated in Figure 1.

Wavenumber [cm^−1^]	Bond Assignment	Reference
853	proline, hydroxyproline, and tyrosine	[10]
935	C–C stretching of proline	[21]
1078	alkyl C–C gauche stretches in lipids	[10]
1265	Amide III and Amide I	[10]
1302	CH_2_ twisting	[10]
1451	CH_2_ and CH_3_ deformation	[21]
1661	Amide III and Amide I	[22]
2860	CH_2_ symmetric stretch of lipids	[11]
2890	CH_2_ asymmetric stretch	[11]
2935	CH_3_ symmetric band	[21]
3010	unsaturated =CH stretch	[11]
3210	water OH-stretching and NH-stretching	[11]

**Table 3:** Confusion matrices for the validated LDA classifiers.

RS		Predicted		
		Tumor	Healthy	
True Label	Tumor	149	22	Sensitivity 0.87
	Healthy	43	89	Specificity 0.67
		Precision 0.78	Negative predictive value 0.80	Accuracy 0.79
OCT		Predicted		
		Tumor	Healthy	
True Label	Tumor	145	57	Sensitivity 0.72
	Healthy	36	108	Specificity 0.75
		Precision 0.80	Negative predictive value 0.65	Accuracy 0.73

**Table 4:** Confusion matrices of the Bayes classification method for the RS, OCT, and combined RS and OCT modalities at the operating points determined by the C-test, S-test, and Youden index.

		Predicted								
		Confirmatory Test (C-Test)			Screening Test (S-Test)			Youden Index		
		Tumor	Healthy		Tumor	Healthy		Tumor	Healthy	
**True label**	**RS and OCT**									
	**Tumor**	33	7	**Sen.** 0.83	36	4	**Sen.** 0.90	33	7	**Sen.** 0.83
	**Healthy**	2	19	**Spec.** 0.90	5	16	**Spec.** 0.76	2	19	**Spec.** 0.90
		**Prec.** 0.94	**NPV** 0.73	**Accu.** 0.85	**Prec.** 0.88	**NPV** 0.80	**Accu.** 0.85	**Prec.** 0.94	**NPV** 0.73	**Accu.** 0.85
	**RS**									
	**Tumor**	12	28	**Sens.** 0.30	36	4	**Sen.** 0.90	39	1	**Sen.** 0.98
	**Healthy**	2	19	**Spec.** 0.90	9	12	**Spec.** 0.57	10	11	**Spec.** 0.52
		**Prec.** 0.86	**NPV** 0.40	**Accu.** 0.51	**Prec.** 0.80	**NPV** 0.75	**Accu.** 0.79	**Prec.** 0.80	**NPV** 0.92	**Accu.** 0.82
	**OCT**									
	**Tumor**	24	16	**Sen.** 0.60	36	4	**Sen.** 0.90	33	7	**Sen.** 0.81
	**Healthy**	2	19	**Spec.** 0.90	8	13	**Spec.** 0.62	4	17	**Spec.** 0.81
		**Prec.** 0.92	**NPV** 0.54	**Accu.** 0.70	**Prec.** 0.82	**NPV** 0.76	**Accu.** 0.80	**Prec.** 0.89	**NPV** 0.71	**Accu.** 0.82

## Data Availability

The data presented in this study are available from the corresponding author upon request.
